# SURVEYING ADULTS’ DESIRE TO KNOW AND PAY FOR A TEST OF DEMENTIA RISK

**DOI:** 10.1093/geroni/igad104.3611

**Published:** 2023-12-21

**Authors:** Alexandra Reed, Andrew Hooyman, Elizabeth Fauth, Jill Love, Sydney Schaefer

**Affiliations:** Arizona State University, Phoenix, Arizona, United States; Arizona State University, Phoenix, Arizona, United States; Utah State University, Logan, Utah, United States; Neurosessments LLC, Tempe, Arizona, United States; Arizona State University, Phoenix, Arizona, United States

## Abstract

Advances in biomarkers of Alzheimer’s disease (AD) and other dementias are promising, but it is also important to characterize the general public’s desire for such biomarkers. It is also important to disentangle the desire to know test results from the desire to disclose the test results with a medical provider/insurance provider. This is particularly important for populations who are at higher risk of developing dementia but who either do not regularly engage with the medical system or who are skeptical of it. The purpose of this study was to survey adults’ desire to know about their short-term risk of developing dementia, and the factors that impact their desire to know. A survey of 326 participants (mean+SD age: 41.9 + 11.9 yrs; 76% White non-Hispanic; 48% F) was completed via Amazon MTurk. Results indicated that 75% of participants would take such a test, but only 12% of them would want their medical/insurance provider to know. Non-White participants (n=73) also reported less desire to share knowledge with a medical/insurance provider, but still wanted to know their risk as much as White participants. Furthermore, the out-of-pocket price that participants were willing to pay for such a test was $100. These data suggest that a diverse set of biomarkers needs to be developed that would enable affordable screening within and outside of a medical context.

